# Upregulation of RIN3 induces endosomal dysfunction in Alzheimer’s disease

**DOI:** 10.1186/s40035-020-00206-1

**Published:** 2020-06-18

**Authors:** Ruinan Shen, Xiaobei Zhao, Lu He, Yongbo Ding, Wei Xu, Suzhen Lin, Savannah Fang, Wanlin Yang, Kijung Sung, Brian Spencer, Robert A. Rissman, Ming Lei, Jianqing Ding, Chengbiao Wu

**Affiliations:** 1 Institute of Neurology, Ruijing Hospital, Shanghai JiaoTong University School of Medicine, 197 Ruijin Er Rd., Shanghai, 200025 China; 2 Department of Neurosciences, University of California San Diego School of Medicine, Room 312 MC-0624,9500 Gilman Drive, La Jolla, CA 92093-0624 USA; 3 Shanghai Institute of Precision Medicine, Shanghai, 200125 China; 4 Department of Neurology, Zhuijiang Hospital, Southern Medical University, Guangzhou, China; 5 San Diego VA Health System, San Diego, CA USA

**Keywords:** Alzheimer’s disease (AD), AD risk factors, Endosomes, Trafficking, RIN3, BIN1, CD2AP, Tau

## Abstract

**Background:**

In Alzheimer’s Disease (AD), about one-third of the risk genes identified by GWAS encode proteins that function predominantly in the endocytic pathways. Among them, the Ras and Rab Interactor 3(RIN3) is a guanine nucleotide exchange factor (GEF) for the Rab5 small GTPase family and has been implicated to be a risk factor for both late onset AD (LOAD) and sporadic early onset AD (sEOAD). However, how RIN3 is linked to AD pathogenesis is currently undefined.

**Methods:**

Quantitative PCR and immunoblotting were used to measure the RIN3 expression level in mouse brain tissues and cultured basal forebrain cholinergic neuron (BFCNs). Immunostaining was used to define subcellular localization of RIN3 and to visualize endosomal changes in cultured primary BFCNs and PC12 cells. Recombinant flag-tagged RIN3 protein was purified from HEK293T cells and was used to define RIN3-interactomes by mass spectrometry. RIN3-interacting partners were validated by co-immunoprecipitation, immunofluorescence and yeast two hybrid assays. Live imaging of primary neurons was used to examine axonal transport of amyloid precursor protein (APP) and β-secretase 1 (BACE1). Immunoblotting was used to detect protein expression, processing of APP and phosphorylated forms of Tau.

**Results:**

We have shown that RIN3 mRNA level was significantly increased in the hippocampus and cortex of APP/PS1 mouse brain. Basal forebrain cholinergic neurons (BFCNs) cultured from E18 APP/PS1 mouse embryos also showed increased RIN3 expression accompanied by early endosome enlargement. In addition, via its proline rich domain, RIN3 recruited BIN1(bridging integrator 1) and CD2AP (CD2 associated protein), two other AD risk factors, to early endosomes. Interestingly, overexpression of RIN3 or CD2AP promoted APP cleavage to increase its carboxyl terminal fragments (CTFs) in PC12 cells. Upregulation of RIN3 or the neuronal isoform of BIN1 increased phosphorylated Tau level. Therefore, upregulation of RIN3 expression promoted accumulation of APP CTFs and increased phosphorylated Tau. These effects by RIN3 was rescued by the expression of a dominant negative Rab5 (Rab5^S34N^) construct. Our study has thus pointed to that RIN3 acts through Rab5 to impact endosomal trafficking and signaling.

**Conclusion:**

RIN3 is significantly upregulated and correlated with endosomal dysfunction in APP/PS1 mouse. Through interacting with BIN1 and CD2AP, increased RIN3 expression alters axonal trafficking and procession of APP. Together with our previous studies, our current work has thus provided important insights into the role of RIN3 in regulating endosomal signaling and trafficking.

## Background

Alzheimer’s disease (AD) is a progressive neurodegenerative disorder that results in memory loss and cognitive impairment [1]. The classical neuropathological hallmarks for AD include Aβ-amyloid-containing neurotic plaques and phosphorylated Tau-containing neurofibrillary tangles (NFT) [2]. In addition, significant synaptic loss, selective neuronal death, neurotransmitter loss and neuroinflammation are also associated with AD pathology [3, 4]. Despite of significant efforts and investments, there are currently no disease-modifying treatments for AD [5–7].

One of the most important lessons learned from these failed clinical efforts is that the cause(s) for AD is far more complex than we have realized. In 2009, apolipoprotein E *(APOE)* that functions in lipid transport, Aβ trafficking, synaptic function, immune regulation, and intracellular signaling [8], was identified as a risk factor for LOAD [9]. More recently, large scale GWAS and a meta-analysis of LOAD have identified more than 20 additional risk factors [10–13]. Identification of these additional risk factors further illustrates the extraordinary genetic complexity of AD.

A significant number of these newly identified loci (PICALM, BIN1, EPHA1, CD2AP, MEF2C, PTK2B, SORL1 and RIN3) that encode products all function significantly in endocytic trafficking and signaling [4]. Given that neurons have the most extraordinary architecture with elaborate dendrites/ axons and will undoubtedly impose a significant need for endocytic processes, it is not surprising that these early endocytic pathways are found to be dysregulated in AD [14–17]. For instance, amyloidogenic cleavage of APP to yield Aβ likely occurs predominantly in the intracellular compartments. Under normal conditions, early endosome, marked by Rab5, is a major site of APP processing by β-secretase (BACE1) to yield the β-cleavage C-terminal fragment (βCTF), which is further processed in late endosomes/trans-Golgi network (TGN) to give rise to Aβ [17–21]. Therefore, trafficking and processing of APP to produce toxic βCTFs/Aβ is intimately regulated by the endocytic pathways.

Although much less is known about the role of RIN3, increasing evidence has pointed to that RIN3 plays an important role in AD. Initial GWAS studies identified a locus (*rs10498633*, G/T), upstream of the RIN3 coding sequences within its enhancer region. It is speculated that this single nucleotide polymorphism (SNP) likely results in increased expression of RIN3 in AD [11]. When comparing extreme AD cases with centenarian controls, the variant effect size for the SLC24A4/RIN3 SNP increased by 4.5-fold, versus 2-folds for APOE-ε4 and 6.5-folds for TREM2 (R74H) variant in comparison to published effect sizes [22]. A more recent case-control study of whole-exome sequencing of 93 sEOAD patients has discovered a missense variant (*rs150221413*, G/T) that results in a substitution of 63 W > C the SH2 (Src Homology 2) of RIN3 [23]. The RIN3^W63C^ variant is likely associated with sEOAD (OR, 4.56; 95% CI, 1.26–16.48; *P* = .02, BP = 0.091) [23]. Furthermore, in a 2017 study of gene methylation profiling of blood and brain samples from 22 AD and 26 normal control subjects (27 males, 21 females), AD samples showed significant group-wide hypomethylation of 7 CpGs located within the 3’UTR of RIN3 (CpG1 p  =  0.019, CpG2 p  =  0.018, CpG3 p  =  0.012, CpG4 p  =  0.009, CpG5 p  =  0.002, CpG6 p  =  0.018, and CpG7 p  =  0.013, respectively) [24]. The effect was specific for RIN3 since other LOAD risk factors (PTK2β, ABCA7, SIRT1, or MEF2C) did not show significant changes in methylation of their promoters. Additionally, a genome wide methylation study of mild cognitive impaired Mexican Americans uncovered significant hypo-methylation in RIN3 and three other genes [25]. Together, these studies have suggested the possibility that increased expression of wildtype RIN3 or expression of the RIN3 variant (W63C) may contribute to AD pathogenesis. However, the underlying mechanism(s) is presently unknown.

RIN3, a guanidine nucleotide exchange factor (GEF), functions as the stimulator and stabilizer for selective members of the Rab5 family (Rab5, 21, 22, 24 and 31) [26, 27]. By cycling between a GDP- bound inactive and a GTP-bound active form, Rab5 regulates endocytosis, intracellular vesicular trafficking [28–30]. However, there is practically nothing known as how increased expression of RIN3 in AD acts on Rab5 to contribute to cellular pathogenesis in AD. We envision that increased expression of RIN3 results in hyperactivation of Rab5 and impairment of endocytic trafficking and signaling. As such, production and accumulation of toxic APP-CTFs is elevated and phosphorylated Tau is increased, thus contributing to neuronal degeneration in AD. Guided by this hypothesis, our current study has demonstrated that RIN3 expression was significantly increased in neurons of APP/PS1 mice; RIN3 interacted with BIN1/CD2AP to regulate APP trafficking and processing; Increased levels of RIN3 significantly impacted endocytic trafficking process and APP cleavage, that may lead to neuronal degeneration in early AD pathogenesis.

## Materials and methods

### Ethical statements

All experiments involving the use of laboratory animals have been approved by the Institutional Animal Care and Use Committees of Shanghai JiaoTong University and University of California San Diego. Surgical and animal procedures were carried out strictly following the NIH Guide for the Care and Use of Laboratory Animals. When possible, every effort was made in our experiments to measure differences between male and female animals.

### Animals

APP/PS1 transgenic animals were obtained from The Jackson Laboratory (number 005864) on the C57BL/6 background. All mice were housed under standard conditions at 22 °C and a 12 h light: dark cycle with free access to food and water. Animal care and handling was performed according to the Declaration of Helsinki and approved by the local ethical committees. Both transgenic APP/PS1 and WT mice in the same littermates were analyzed. For mRNA and protein assays of brain tissues, only male mice of different ages (0.25, 1, 3, 6, 9, 10 months) were used. At least 4 mice in each group*.*

### Quantitative PCR

Total RNAs from either mouse tissues or cultured neurons were extracted using the Trizol method according to the manufacturer’s protocol. The RNA samples were reverse-transcribed into cDNA using the PrimeScript first Strand cDNA Synthesis Kit (Takara). Quantitative real-time PCR method was used to detect the expression of RIN3. The experimental reaction consisted of 40 cycles of 95 °C for 30 s, 60 °C for 5 s, and 72 °C 30 s, the PCR products of the target gene and the internal control gene (GAPDH) were detected and quantitated by fluorescent SYBR Green (Takara) using an ABI7500 real-time PCR machine. Threshold cycle values (Ct) were generated at each cycle during a run. Relative quantification of mRNA expression was calculated by the ΔCt method after adjusting the levels to the corresponding internal GAPDH and normalized against WT samples.

### Chemicals, reagents, and antibodies

HBSS, GlutaMAX, Neurobasal, B27, Lipofectamine 2000, donkey anti-rabbit IgG–Alexa 488, donkey anti-goat IgG–Alexa 568 conjugates were from Invitrogen. DMEM high glucose was from Mediatech. FBS was from Phoenix Research Products. HEPES, poly-l-lysine, AraC and bisbenzimide H (Hoechst 33258) were from Sigma-Aldrich.

C15 antibody used to detect full length APP and APP C-terminal fragments was a gift from Dr. Edie Koo (UCSD). Tau PHF-1 (ser396/404) antibody was a gift from Peter Davis (Albert Einstein University). Rabbit anti-Tau (Ser^396^) polyclonal Antibody from GenScript (Cat# A01387). Mouse anti-GFP IgGs (sc-9996), rabbit anti-Rab5B IgGs (sc-598), mouse anti-Rab7 IgGs(sc-10,767) and mouse anti-Rab11 IgGs (sc-166,912) were from Santa Cruz Biotechnology Inc. Rabbit anti-RIN3 IgGs (ab64838) were from Abcam. Rabbit anti-BIN1 IgGs (PA5–28391) and Rabbit anti-CD2AP IgGs (PA5–34687) were from ThermoFisher. Rabbit-anti-Flag IgGs (393–107) were from ABR Affinity Bioreagents Inc. Mouse anti-MAP2 IgGs (MAB378) were from Millipore. Mouse anti-GAPDH IgGs (GT239) were from GeneTex. Goat anti-rabbit or anti-mouse IgG–HRP were from Jackson ImmunoResearch Laboratories Inc.

### Plasmids and siRNAs

The APP-mCherry and Rab5^S34N^-mCherry expression vectors were described before [21]. The full-length cDNA of RIN3 was generated by PCR using a human peripheral blood mononuclear cells (PBMCs) cDNA library. Neuronal isoform cDNA of BIN1 and full-length of CD2AP were synthesized by Biosune Inc. All cDNA was cloned into either pEGFP-N1 or pcDNA3.4-flag vector. RIN3-ΔSH2(Δ63–158 aa), RIN3-ΔRH(Δ587-732aa), RIN3-ΔVps9(Δ703-846aa), RIN3-ΔRas(Δ877-963aa) were generated from full-length RIN3 and cloned into pEGFP-N1 vector using standard molecular cloning techniques. BACE1-mCherry construct is a gift from Dr. Uptal Das (UCSD). All expression vectors were verified by sequencing.

### Primary neuron culture and transfection

Basal forebrain cholinergic neurons (BFCNs) were cultured from WT and APP/PS1 transgenic mouse E18 embryos as described previously [21, 31]. Briefly, basal forebrains were dissected, dissociated, and resuspended in Neurobasal with 10% FBS, B27, GlutaMAX, and 50 ng/ml NGF and plated for 4 h. Maintenance medium (Neurobasal with B27, GlutaMAX, and 50 ng/ml NGF) was added to the cell culture and incubated for 24 h before being replaced with antimitotic medium (maintenance medium plus 1 μM AraC) for 12 h, followed by switching to maintenance medium. For transfection, 0.5 μg DNA and Lipofectamine 2000 were used. An ~ 10% transfection efficiency was achieved. Cells were imaged 24–48 h after transfection.

### Cell culture and transfection

HEK293T cells or PC12M cells were cultured in DMEM high glucose with 10% FBS [32]. Cells were plated into 12-well/6-well culture plates 24 h, and the confluency of the cells were about 80% prior to transfection. For plasmid transfection, Lipofectamine 2000 were used according to the manufacture’s protocol. siRNAs were transfected with Lipofectamine iRNAmax. An approximately 60–70% transfection efficiency was achieved. Cells were harvested 24–48 h after transfection.

### Immunofluorescence

BFCNs or PC12M cells cultured on PLL-coated glass coverslips were fixed in 4% PFA for 15 min at 37 °C and were permeabilized and blocked with 0.15% Triton X-100 in 3% donkey serum in PBS for 15 min. Cells were incubated with primary antibodies (1:100 for Rab5, Rab7, Rab11; 1:200 for Flag, 1:50 for BIN1 and 1:50 for CD2AP, 1/200 for MAP2) overnight at 4 °C. Cells were rinsed and incubated with secondary antibody for 1 h at room temperature. Nuclei were stained with Hoechst 33258 (0.1 μg/ml) for 10 min. All images were collected with a Confocal Laser Scanning Microscope (Leica).

### Co-immunoprecipitation and mass spectrum

HEK293T cells cultured in 6 cm dish were transfected with RIN3-Flag vector and were harvested 48 h post transfection. Cells were solubilized with lysis buffer (150 mM NaCl, 50 mM Tris-HCl pH = 7.5, 1% (w/v) Triton and 1 mM phenylmethanesulfonyl fluoride). Protein lysates were extracted and incubated with anti-DYKDDDDK G1 affinity Resin (GenScript, L00432) and incubated at 4 °C for 30 min. The samples were centrifuged at 1000 rpm for 1 min and the supernatant was discarded. The resin was washed three times with washing buffer containing 150 mM NaCl, 50 mM Tris-HCl (pH = 7.5). Proteins were eluted from the resin with DYKDDDK peptide. Mass spectrometry was performed to analyze compositions of the RIN3-containing protein complexes.

### Co-immunoprecipitation and Western blotting

RIN3-GFP was co-transfected with either BIN1-Flag or CD2AP-Flag into HEK293T cells cultured in 6 cm dishes. The cells were harvested after 24 h post transfection, washed twice with PBS and solubilized with 500 μl of lysis buffer as described above. Protein lysates were extracted and incubated with either anti-Flag IgGs (0.5 μg) or control IgG (0.5 μg) overnight at 4 °C. 40 μl protein A/G agarose beads were added and incubated for 2 h at 4 °C. Samples were centrifuged at 1000 rpm centrifuge and supernatant was discarded. The resin was washed three times with washing buffer. Proteins were eluted from the resin with 40 μl SDS loading buffer and boiled at 95 °C for 5 min. Immunoblotting was performed with anti-GFP antibody and Anti-Flag antibody.

### Yeast two-hybrid assay

Yeast two-hybrid assays were performed as previously described [33]. Colonies containing the L40 strain harboring pBTM116 and PACT2 (Clontech) fusion plasmids were selected on Leu-Trp plates. β-Galactosidase activities were measured by a liquid assay.

### Live cell imaging, axonal transport of APP and BACE1

APP-mCherry, BACE1-mCherry or mCherry was transfected into mouse E18 primary cortical neurons at DIV4–5. For co-transfection, EGFP, RIN3-GFP, BIN1-GFP and CD2AP-GFP constructs were co-transfected with mCherry or APP-mCherry or BACE1-mCherry and incubated overnight. Time-lapse images were taken at 1 frame/3 s for a total of 90 s. 24 h post transfection, time-lapse images were captured at 1 frame/sec for a total of 2 min per series using a Leica DMI6000B inverted microscope, which is equipped with an environmental chamber (37 °C) as described previously [21, 34]. Both fluorescent images and DIC images were also captured for analysis. Generation of kymographs and analysis of transport were performed using ImageJ [21, 34].

### Statistical analysis

All experiments were repeated at least 3 times independently. Data represent mean ± SEM. Statistical analyses and calculation of *p* values were performed using Prism6(GraphPad Software, La Jolla, CA); Standard *t* test was used for pairwise comparisons and two-way ANOVA for multiple comparisons. *p* values less than 0.05 were considered statistically significant, and *p* values less than 0.01 were considered statistically highly significant.

## Results

### APP/PS1 mice show increased RIN3 expression and early endosome enlargement

Given that GWAS analysis and methylation profile study revealed that expression of RIN3 is likely upregulated in AD [13, 24], we decided to investigate if this was also true in a mouse model of AD. We elected to measure both RIN3 mRNA and protein level in the APP/PS1 mouse model of AD. The APP/PS1 model expresses a chimeric mouse/human amyloid precursor protein (Mo/HuAPP695swe) and a mutant human presenilin 1 (PS1-dE9). Both APP695swe and PS1-dE9 mutations are associated with early-onset Alzheimer’s disease [35–37].

**Fig. 1:**
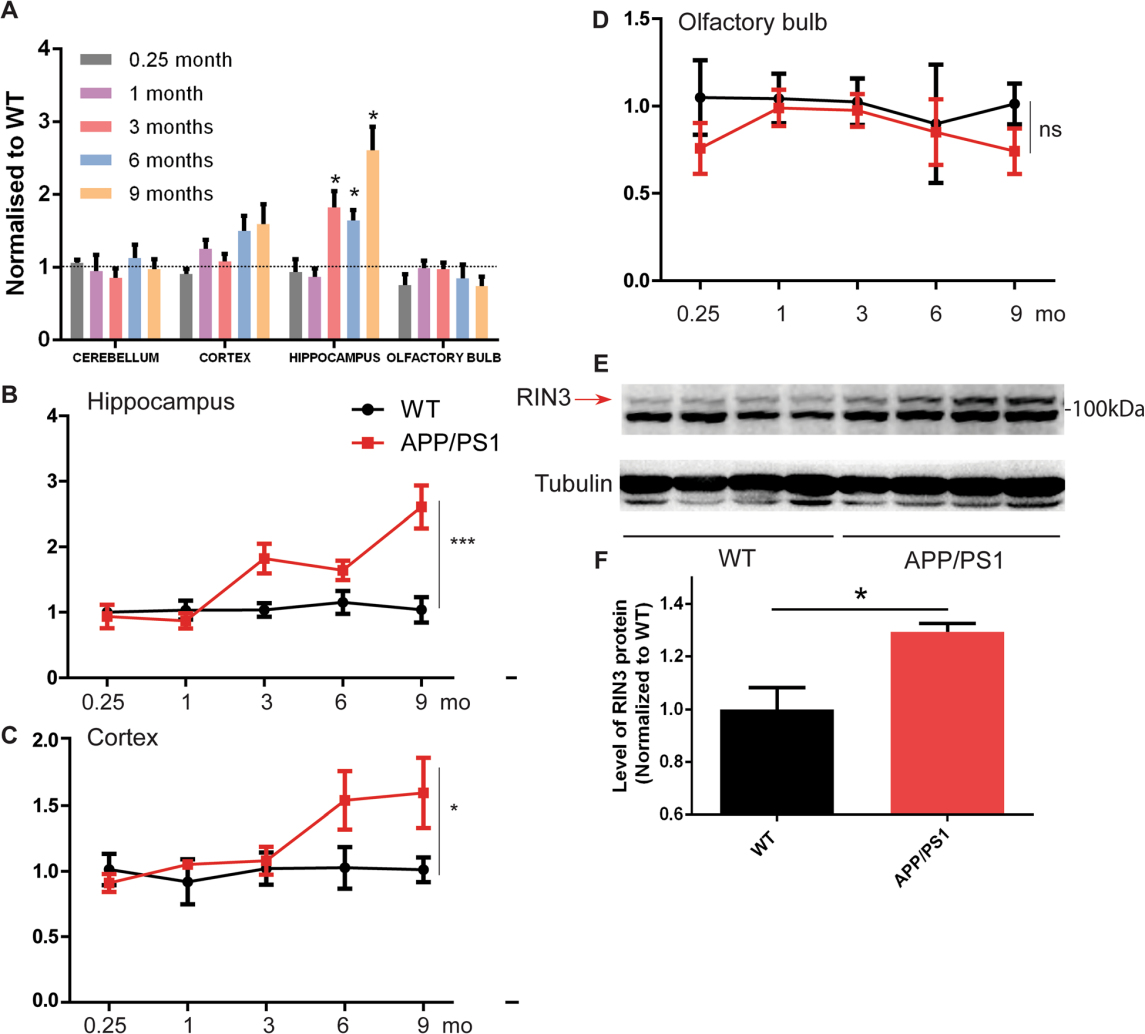
RIN3 is upregulated in APP/PS1 mice hippocampus at 3 months. **a**: RIN3 mRNA was measured in cerebellum, cortex, hippocampus, olfactory bulb of APP/PS1 and WT at different ages as indicated (*n* = 3 for all age groups). Hippocampus (**b**), cortex (**c**) and olfactory bulb (**d**) were further examined in great details. Data are mean ± SEM, *p* < 0.05 (*), *p* < 0.001 (***), n.s: non-significant, two-way ANOVA; **e**. RIN3 protein levels were measured by SDS-PAGE immunoblotting with a specific antibody against RIN3, with tubulin as a loading control. The intensity of RIN3 bands was quantitated using BioRad-ImageLab. *P* < 0.05 (*), standard t-test

We first used quantitative PCR (qPCR) to measure the mRNA level in different brain region of APP/PS1 mice from 1 week old to 9 months of age and compared the measurements with their age-gender matched wild type (WT) mice. As shown in Fig. 1a, APP/PS1 mice showed no significant changes in RIN3 mRNA level in either the cerebellum or the olfactory bulb (Fig. 1a, d). However, RIN3 mRNA was significantly increased in both the cortex and the hippocampus (Fig. 1a-c), two brain regions that are markedly impacted in AD. The increase in RIN3 mRNA in the hippocampus became significantly evident as early as at 3 months of age (Fig. 1b). Similarly, starting at 3 months of age, RIN3 mRNA level in the cortex increased and reached a significant difference at 6 months of age (Fig. 1c). These results are significant since these mice do not develop β-amyloid deposits in their brain until they reach 6 to 7 months of age. We thus conclude that upregulation of RIN3 precedes β-amyloid deposits in the APP/PS1 AD mice.

To define if expression of RIN3 at the protein level was also increased in APP/PS1 mice, we used immunoblotting to measure RIN3 protein in the hippocampus of 3 months old male APP/PS1 mice. Our results confirmed that the RIN3 protein level (Fig. 1e, f) showed an increase in these mice as compared to age-matched WT littermates. Taken together, our results have demonstrated that expression of RIN3 is increased in early development of APP/PS1 mice, well preceding the appearance of Aβ plaques in these mice.

**Fig. 2:**
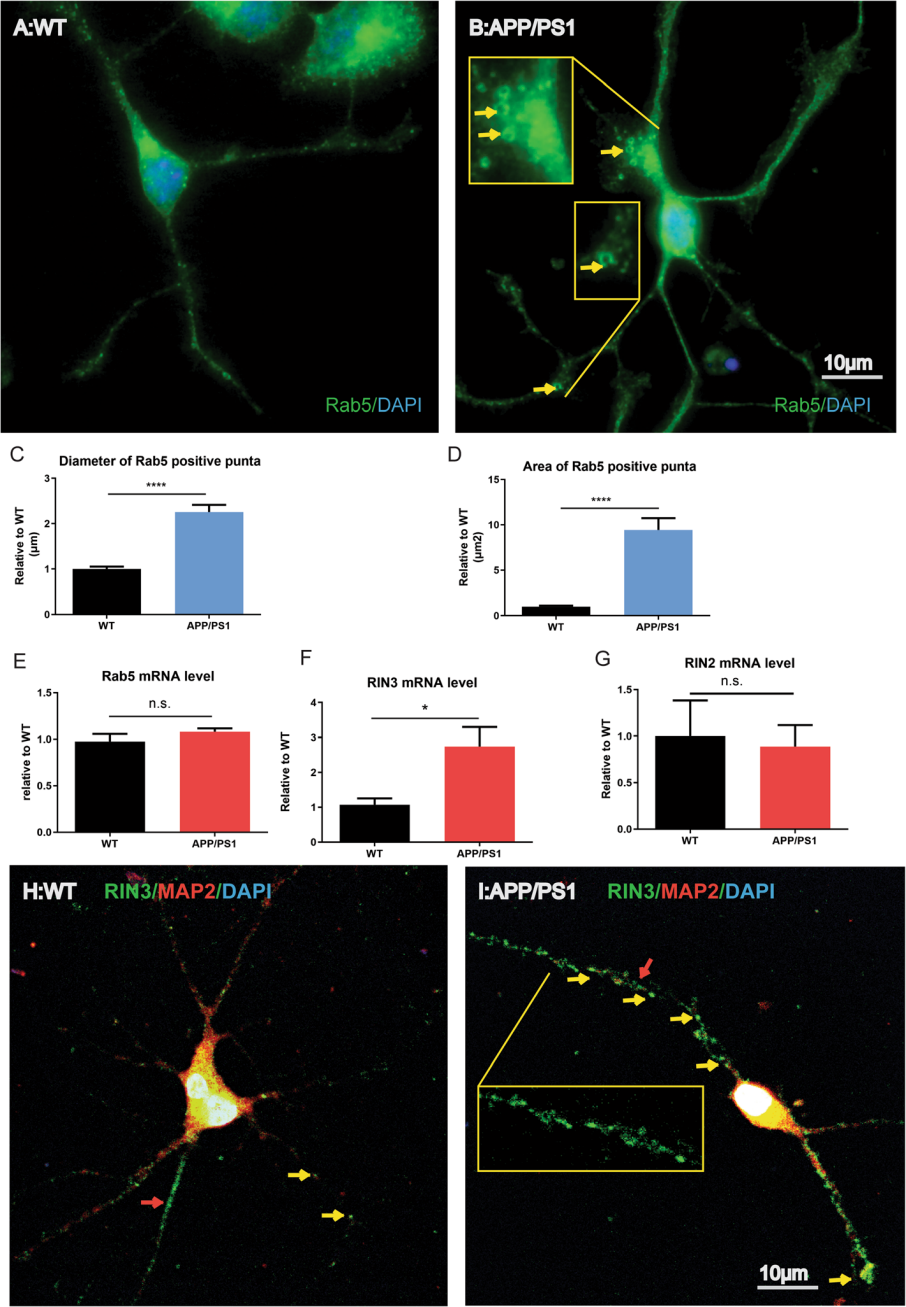
RIN3 upregulation in APP/PS1 BFCNs induce enlarged early endosome. E18 BFCNs of WT (**a**) and APP/PS1 (**b**) were cultured and fixed at DIV4 for immunostaining with Rab5 antibody. Nuclei were stained with DAPI. Rab5 positive vesicles were measure by diameter (**c**) and size (**d**) using ImageJ (in blue bar graph). The mRNA level for Rab5 (**e**), RIN3 (**f**) and RIN2 (**g**) were quantitated using qPCR (in red bar graph). Two tail T-test was used. * p < 0.05 and ***p < 0.001. As in A and B, E18 BFCNs of WT (**h**) and APP/PS1 (**i**) were also co-stained for RIN3 and MAP2. The inset in **I** shows enlarged RIN3 puncta. Representative images are shown

BFCNs are believed to have some of the longest axons [38–44]. These large axonal projections make BFCN neurons extremely vulnerable, it is thus not surprising that basal forebrains degenerate early in AD [39]. Previously, we showed that Rab5 endosomes were enlarged in BFCNs when APP was overexpressed [21]. We cultured E18 BFCNs from APP/PS1 and their WT control embryos. We first stained for Rab5 using a specific antibody. Our results showed that the size of Rab5 vesicles were indeed increased in APP/PS1 neurons as compared to WT neurons (Fig. 2b versus a). This became very evident by the quantitation of both the diameters (Fig. 2c) and areas of Rab5 endosomes (Fig. 2d). However, qPCR analysis showed no increased in Rab5 at the message RNA level (Fig. 2e).

To define if RIN3 expression was also increased in BFCN neurons that tracked early endosomal pathologies in cultured APP/PS1 BFCNs, we also measured the RIN3 mRNA level by qPCR. Intriguingly, APP/PS1neurons showed a significant increase in RIN3 expression (Fig. 2f). To determine if the increase was specific for RIN3, we also measured the expression level of RIN2(Fig. 2h), another member of the RIN family. Our results showed no changes in the RIN2 mRNA level. Therefore, RIN3 is selectively upregulated in cultured BFCN neurons of APP/PS1 mice. Since RIN3 is a GEF for Rab5, increased RIN3 expression likely contributes to enlarged Rab5 early endosomes in cultured BFCNs from APP/PS1.

To further define the subcellular localization of RIN3-Rab5 vesicles, we co-stained BFCN for RIN3 [21] and MAP2, a somal/dendritic marker using specific antibodies. As shown in Fig. 2h, i, RIN3 positive puncta were present in soma and dendrites (both are immunoreactive to anti-MAP2 antibody) (Fig. 2h, i). RIN3 puncta were also seen in processes that were negative for MAP2. i.e. axons (indicated by arrows) (Fig. 2h). Further, the size of RIN3 puncta in APP/PS1 BFCNs appeared to be enlarged in comparison to WT neurons (Fig. 2i versus h, also see the insert). Together, our results have revealed that a selective increase in RIN3 expression may induce enlargement of Rab5-early endosomes in cultured E18 mouse BFCNs of APP/PS1 mice.

### RIN3 interacts with two other AD risk factors, CD2AP and BIN1

**Fig. 3:**
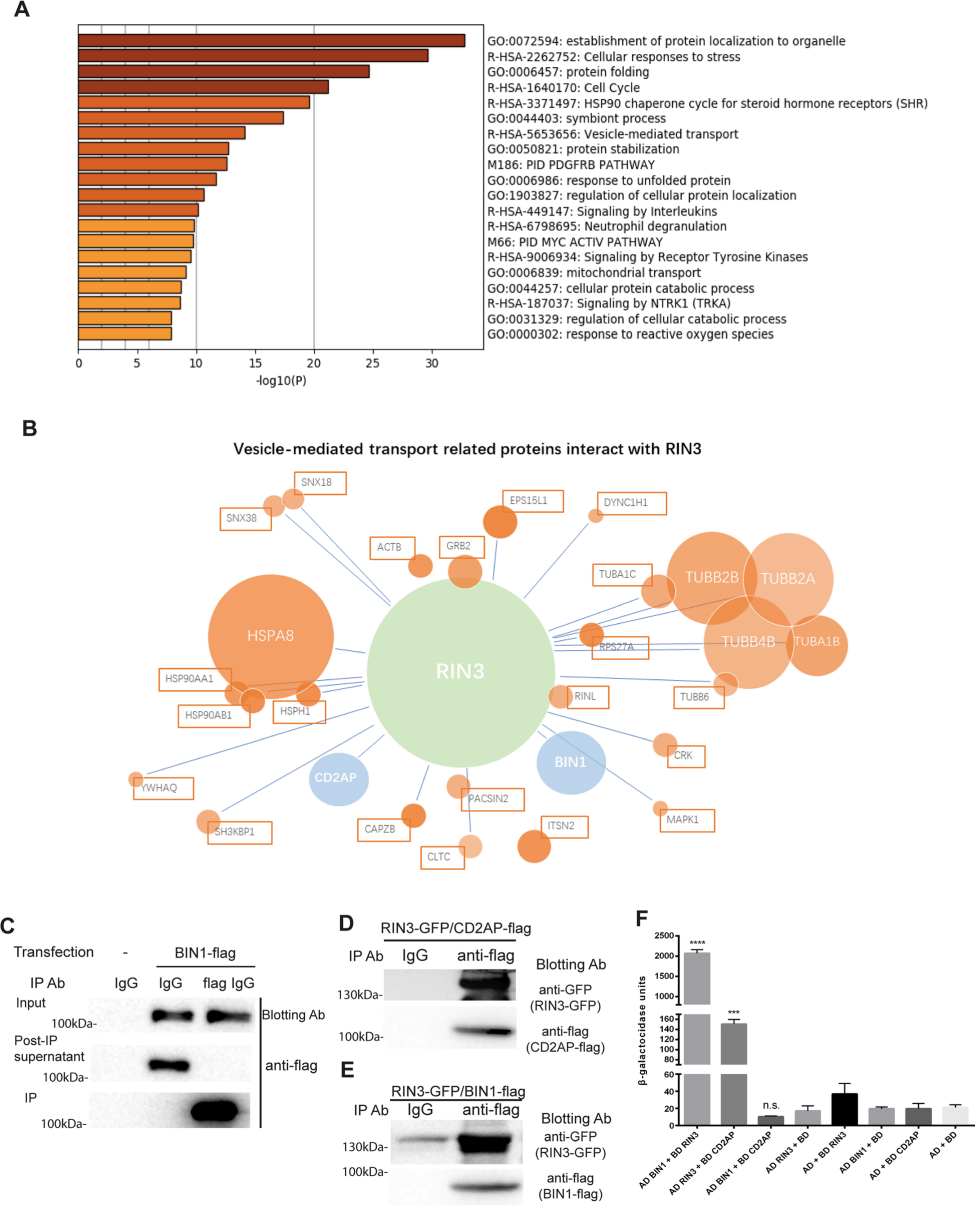
BIN1 and CD2AP interact with RIN3. RIN3-flag was expressed and purified from HEK293t cells, then MS was used to identify and define RIN3 interactomes. **a**: A set of 380 RIN3-interacting proteins were detected, and a GO analysis was conducted. The RIN3 interactomes were divided into 20 categories with respect to different cellular function. **b**: A protein interaction network highlighting the RIN3-interactome that regulate endocytic/vesicular transport, based on 28 highly enriched proteins (PSM value ≥5). The circle size indicated the degree of enrichment. RIN3 was in green, LOAD risk factors were in blue. Interaction between RIN3 and CD2AP/BIN was analyzed by co-IP. HEK293 cells were transfected with indicated vectors. Cell lysates were incubated with either control IgG (IgG) and anti-flag IgG. Untransfected cells were used as a control. In **c**, the cell lysate inputs, the post-IP supernatant and the IP complexes were analyzed by SDS-PAGE/immunoblotting with indicated antibody. In **d**, RIN3-GFP was co-IP with CD2AP-flag; In **e**, RIN3-GFP was co-IP with BIN1-flag. For both D, E, a control IgG (IgG) was used as a negative control. RIN3-GFP was blotted with a GFP antibody, an anti-flag antibody was used to detect CD2AP-flag (**d**) or BIN1-flag (**e**). **e** Yeast two hybrid assay was performed, each group had three replicants. Standard t-test, **** stands for *p* < 0.0001, *** stands for p < 0.001, n.s. stands for *p* > 0.05

To further understand the biological function of RIN3, we expressed a flag-tagged RIN3 construct in HEK293T cells. RIN3-containing protein complexes were purified and analyzed by Mass Spectrometry to identify the RIN3 interactome. A total of 380 proteins were positively identified to interact with RIN3. Followed by a GO analysis, the RIN3 interactome was divided into 20 groups (Fig. 3a). The RIN3-interactome is highly enriched of proteins (Peptide Spectrum Match, PSM value≥5) that mediate vesicle transport. This is illustrated by a protein interaction network (Fig. 3b).

As expected, many of these RIN3-interacting proteins have been implicated to regulate endocytosis and intracellular vesicular trafficking. Interestingly, our analysis has uncovered that two other AD risk factors were highly associated with RIN3; BIN1 (bridging integrator 1) (PSMs = 22, coverage = 38% and detected peptides = 15) and CD2AP (CD2-associated protein) (PSMs = 21, coverage = 30% and detected peptides = 17) (Supplementary Table 1). Thus, RIN3 likely forms a complex with BIN1/CD2AP.

To confirm the interaction, we conducted co-immunoprecipitation (IP) assay. We co-transfected RIN3-GFP with either CD2AP-flag or BIN1-flag into HEK293T cells. An anti-flag antibody was used to immunoprecipitated CD2AP or BIN1. To ensure the pulldown efficiency of the anti-flag antibody, we assayed the cell lysate inputs, the post-IP supernatant and the IP products. As shown in Fig. 3c, protein lysates from cells transfected with the BIN1-flag construct, but not from the un-transfected cells, gave rise to the expected BIN1-flag protein as recognized by the anti-flag antibody by immunoblotting (Fig. 3c, top panel). In the post-IP supernatant, the BIN1-flag product was still detected in samples that incubated with a control IgG (IgG), but the same band was no longer seen in samples incubated with anti-flag (Fig. 3c, middle panel). As expected, BIN1-flg was only present in the pulldowns with anti-flag but not with the control IgG (Fig. 3c, bottom panel). These data have demonstrated a great efficiency of the anti-flag antibody in IP assays. We then performed the co-IP experiments. Our analysis of the IP complexes has showed that RIN3-GFP was co-immunoprecipitated when co-expressed with either CD2AP-flag (Fig. 3d) or with BIN1-flag (Fig. 3e). Our data are evidence that BIN1 and CD2AP both interact with RIN3. The interaction between RIN3 and BIN1/CD2AP was further confirmed in yeast two-hybrid assays (Fig. 3f). Significantly, no interaction between BIN1 and CD2AP was detected in these assays (Fig. 3f). These results have thus demonstrated that RIN3 directly interacts with BIN1 and CD2AP either simultaneously or separately.

**Fig. 4:**
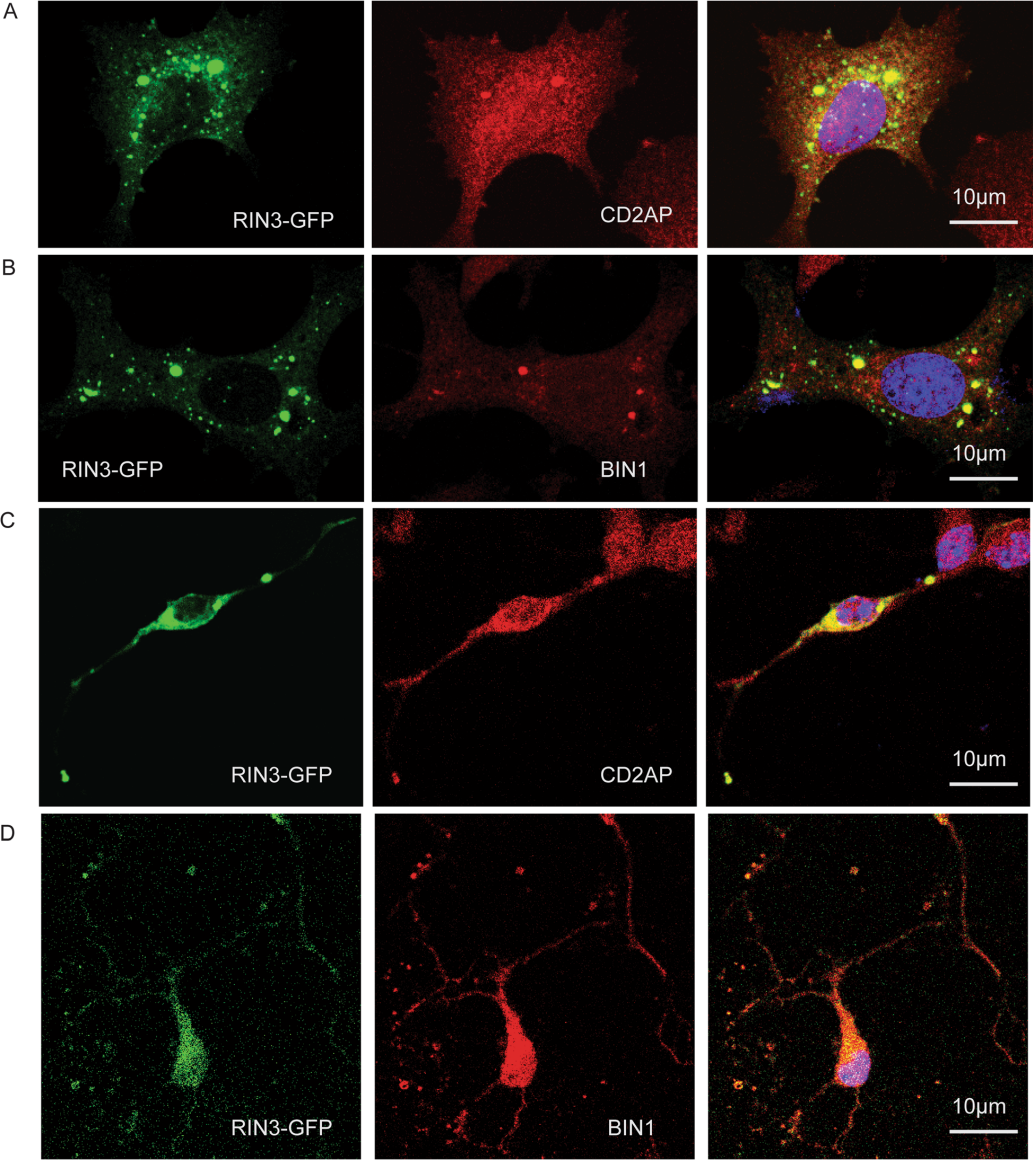
RIN3 colocalizes with CD2AP and BIN1. PC12M cells (**a** and **b**) and primary cortical neurons (**c** and **d**) were transfected with RIN3-GFP, followed by immunostaining for CD2AP (**a**, **c**) or BIN1 (**b**, **d**) or CD2AP using specific antibodies. Yellow color denotes colocalization. Representative images are shown

To further corroborate our IP data that RIN3 interacted with BIN1 and CD2AP, we transfected RIN3-GFP in PC12 cells (Fig. 4a, b) and mouse E18 embryonic primary neurons (Fig. 4c, d). We then used specific antibodies against BIN1 or CD2AP to detect the subcellular distribution of endogenous BIN1 and CD2AP with respect to RIN3-GFP by immunostaining. Of note, we consistently observed enlarged vesicles following expression of RIN3-GFP. Furthermore, both endogenous BIN1 and CD2AP were recruited to these vesicles and colocalized with RIN3(Fig. 4a, b). In primary cultured neurons, RIN3-BIN1 and RIN3-CD2AP puncta were mostly seen in processes (axons and dendrites), but not in cell bodies (Fig. 4c, d). Consistent with previous reports [26, 45, 46], these findings suggest that the RIN3-BIN1-CD2AP complex is likely involved in mediating intracellular vesicular sorting and trafficking.

Structurally, RIN3 has a Src homology 2 (SH2) domain, a proline rich domain (PRD), a RIN-homology (RH) domain, a Vps9 (vacuolar protein sorting-associated protein 9) and a Ras-association (RA) domain [26, 46]. Previous studies have shown that BIN1 and CD2AP interacted with RIN3 through their SH3 domain [26, 47]. We performed further immunofluorescence to define the domain(s) of RIN3 was responsible for its interaction with BIN1 and CD2AP. We selectively deleted each of the following domains: SH2, RH, Vps9, or Ras from RIN3 and constructed RIN3-ΔSH2-GFP, RIN3-ΔRH-GFP, RIN3-ΔVps-GFP, RIN3-ΔRas-GFP vectors, respectively (Figure S1). We then transfected each of these constructs with BIN1-flag or CD2AP-flag into PC12M cell line. Our data showed that deletion of each of these four domains had no effect on the interaction between RIN3 and BIN1 or between RIN3 and CD2AP, since BIN1-flag, CD2AP-flag or Rab5 were still colocalized with RIN3-ΔSH2-GFP, RIN3-ΔRH-GFP, RIN3-ΔVps-GFP and RIN3-ΔRas-GFP (Figure S1). However, deletion of the SH2 or the RH domain did result in diffused localization for RIN3, BIN1, CD2AP and Rab5 throughout the cytoplasm. These results suggest that the SH2 or the RH domain is required for targeting of RIN3 along with BIN1, CD2AP to Rab5 endocytic vesicles. We therefore conclude that either the N-terminal SH2 domain or the PRD domain of RIN3, or both, are likely required for mediating the interaction between RIN3 and BIN1 and CD2AP and target these two proteins to early endocytic vesicles. Taken together with our biochemical, genetic and cellular studies, our results suggest that the three AD risk factors: RIN3, BIN1 and CD2AP may form a functional complex to impact endocytic sorting and trafficking.

### Upregulation of RIN3 induces recruitment of CD2AP and BIN1 to early endosomes

**Fig. 5:**
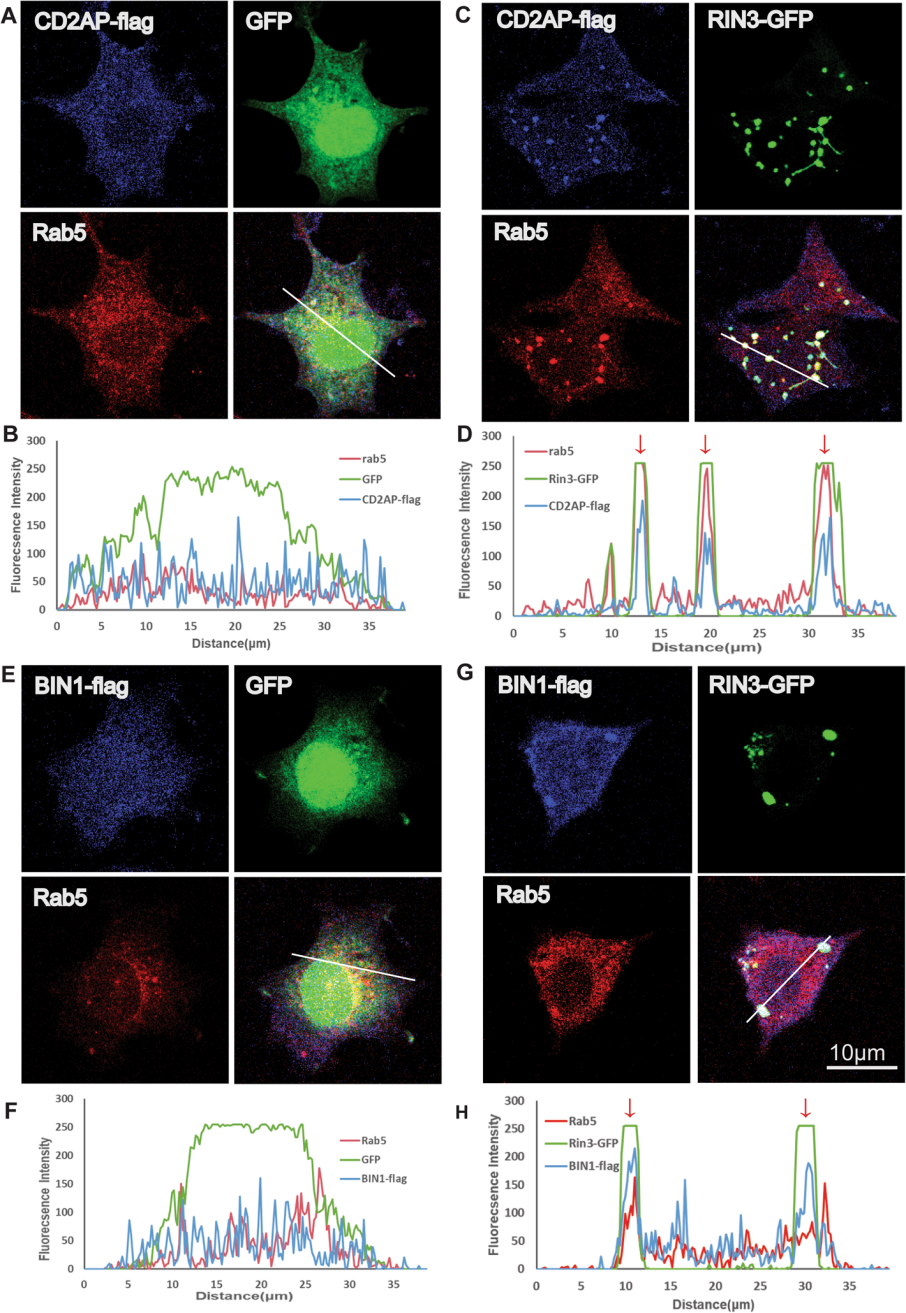
CD2AP and BIN1are recruited to early endosome by RIN3. PC12M cells expressing GFP/CD2AP-flag (**a**, **b**), RIN3-GFP/CD2AP-flag (**c**, **d**), GFP/BIN1-flag (**e**, **f**), RIN3-GFP/BIN1-flag (**e-h**) were immune-stained for Rab5 with a specific antibody. Representative images are shown. Colocalization analysis was performed by imageJ by measuring fluorescence intensity alongside the drawn line. The three different colors peak at the same position If the three proteins were colocalized (**b**, **d**, **f**, **h**)

We next investigated the potential impact(s) of increased expression of RIN3 on BIN1 and CD2AP with respect to Rab5. BIN1 has been shown to be involved in the formation of recycling endosomes from early endosomes [11]. CD2AP is an adaptor that stabilizes internalized receptors in early endosomes, as such, either to prevent receptors from being degraded or to facilitate the transmission of certain signals [45, 48]. Based on our findings that RIN3 interacted with both CD2AP and BIN1, RIN3 was localized to early endosomes, we thus postulated that RIN3 recruited CD2AP and BIN1 to early endosomes. To investigate this, we examined the cellular localization of CD2AP, BIN1 with or without co-expression of RIN3. In the absence of RIN3 expression, the signals for CD2AP-flag (Fig. 5a, b) and BIN1-flag (Fig. 5e, f), as revealed by IF, were mostly diffused across the cytoplasm in PC12 cells, with little or no presence on Rab5-early endosomes (Fig. 5a, e). By co-transfection with RIN3-GFP, the signals for CD2AP (Fig. 5c, d) and BIN1 (Fig. 5g, h) became colocalized with Rab5, but not with Rab7 (Figure S2) or Rab11 (Figure S3). Of note, colocalization of the three different colors is indicated by the appearance of peaks of fluorescence intensities at the same position(s), as shown in Fig. 5d and h (red arrow heads). Likewise, non-colocalization is revealed by the three peaks that are randomly distributed as in Fig. 5b and f. These results provide further evidence that increased expression of RIN3 recruits CD2AP and BIN1 specifically to early endosomes, but not to late endosomes or recycling endosomes.

### Role of RIN3, BIN1 and CD2AP in APP and BACE1 trafficking

**Fig. 6:**
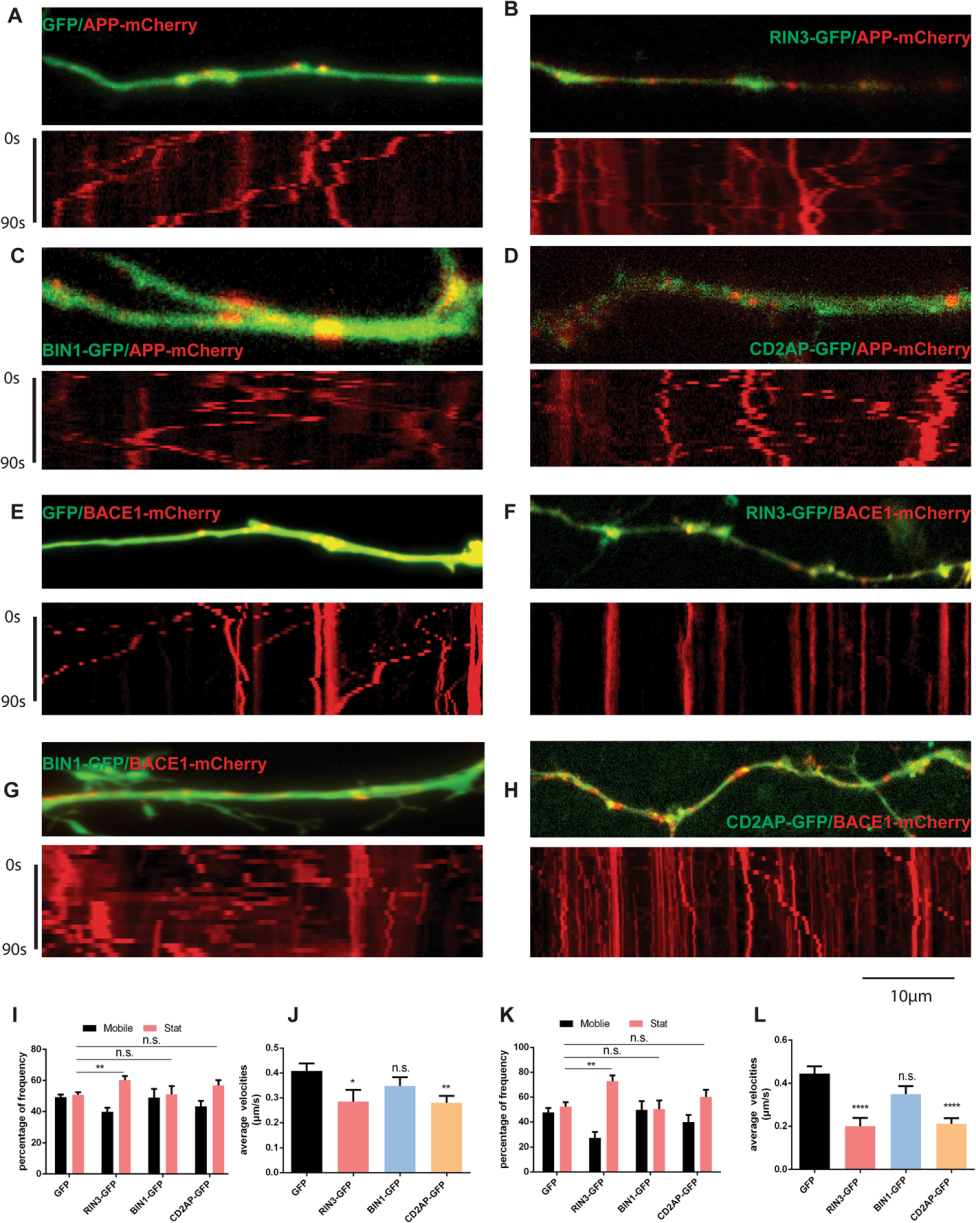
RIN3 and CD2AP inhibited transport of APP and BACE1 in primary cortical neurons. E18 mouse cortical neurons were cultured in microfluidic chambers and were co-transfected with the indicated expression vectors. Live imaging was performed as described in the Methods section. Representative images of axons and corresponding kymographs are shown in **a-h**. The percentiles of mobile versus stationary vesicles (stat) (**i**, **k**) and average velocities (**g**, **l**) for APP-mCherry and BACE1-mCherry in axons were quantitated. Data represent mean ± SEM of at least 3 independent experiments. All *p-*values were calculated using 1-way ANOVA. *p* < 0.05 (*), *p* < 0.01(**), *p* < 0.0001(****), *p* > 0.05 (n.s.), standard t-test

RIN3, BIN1 and CD2AP have all been implicated in regulating endocytic trafficking. To define their role in axonal transport, we expressed APP-mCherry or BACE1-mCherry in mouse E18 primary cortical neurons either alone or with RIN3-GFP, BIN1-GFP and CD2AP-GFP, respectively. As a control, we first examined APP-mCherry- or BACE1-mCherry in axons of neurons co-transfected with EGFP. The signals for EGFP were diffused while the signals for APP-mCherry or BACE1-mCherry appeared as red puncta (Fig. 6, a, e, also Figure S4A). Similar results were found in axons of neurons co-transfected with BIN1-GFP and APP-mCherry or BACE1-mCherry (Figure S4A, B). In contrast, neurons that express either RIN3-GFP or CD2AP-GFP showed distinct features in their axons from those of expressing BIN1-GFP. The signals for RIN3-GFP and CD2AP-GFP were overlapped significantly with either APP or BACE1 puncta (Figure S4B, C).

To ask whether or not overexpression of RIN3, BIN1 or CD2AP impacted axonal transport, we examined axonal transport of APP-mCherry (Fig. 6, a, c, e, g) and BACE1-mCherry (Fig. 6, b, d, f, h) by live imaging 24 h after transfection. Time-lapse image series were captured, and kymographs were generated; representative images with corresponding kymographs for each condition are shown (Fig. 6). When compared with EGFP transfected neurons, cortical neurons expressing either RIN3-GFP or CD2AP-GFP showed a significant reduction in both the percentile of mobile puncta and the average transport velocities for APP and BACE1 (Fig. 6 i-l). In RIN3-GFP transfected neurons, the average transport velocities of APP-mCherry and BACE1-mCherry particles were significantly reduced by approximately 30.2% (*p* = 0.0222) and 55.3% (p<0.0001), respectively; And the percentiles of moving puncta were also significantly reduced by 9.54% (*p* = 0.0083) and 20.5% (p = 0.0083), respectively (Fig. 6i-l). The average moving velocities of APP-mCherry and BACE1-mCherry particles seen in CD2AP-GFP transfected neurons were also reduced by 31.4% (*p* = 0.002) and 52.7% (*p* < 0.0001) while the decrease in the percentiles of mobile puncta were not significant (*p* = 0.2954 and *p* = 0.1435) (Fig. 6i-l). Neither the moving velocities nor the percentile of mobile puncta for APP-mCherry and BACE1-mCherry exhibited significant difference from either the control or from BIN1-GFP expressing neurons (*p* = 0.192 and *p* = 0.0797) (Fig. 6i-l).

### Role of RIN3/BIN1/CD2AP in APP processing and tau phosphorylation

**Fig. 7:**
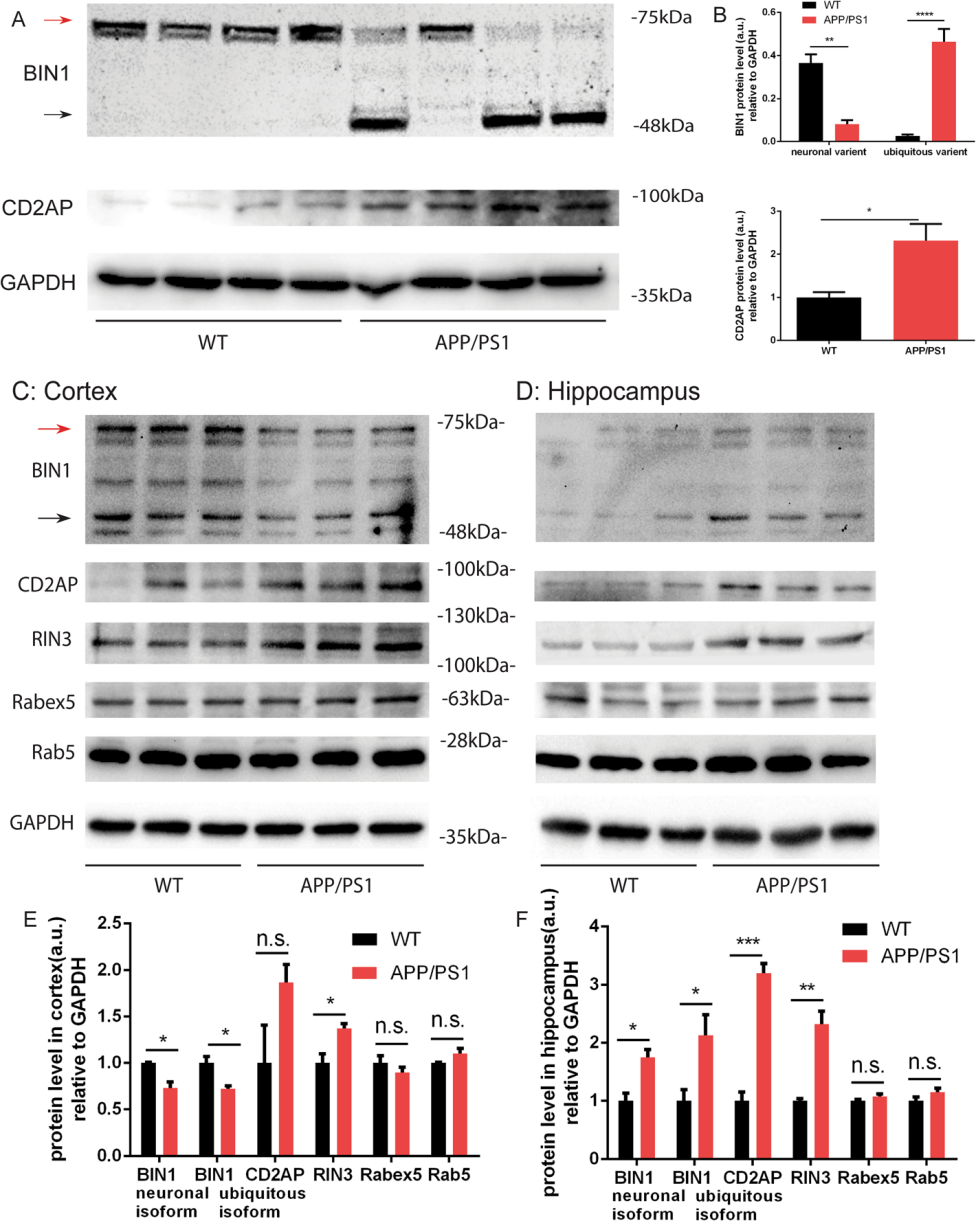
BIN1 and CD2AP expression level in APP/PS1 mice. 3-month-old APP/PS1 mice and age-matched WT mice were dissected, lysates were immunoblotted for BIN1 and CD2AP (**a**). The protein levels were normalized against GAPDH as a loading control (**b**). In Cortex (**c**) and hippocampus (**d**) were also extracted from10-month-old APP/PS1 and age-matched WT mice. Protein lysates were used to probe for BIN1, CD2AP, RIN3, Rabex5 and Rab5 using indicated antibodies by SDS-PAGE/immunoblotting (**c** and **d**), The intensity of bands was quantitated using BioRad-ImageLab. The respective protein levels were normalized against GAPDH as a loading control (**e**, **f**). *p* < 0.05 (*), *p* < 0.01(**), *p* < 0.0001 (****), standard t-test

Our current study has suggested a strong possibility that RIN3 may function as a scaffold to tether BIN1 and CD2AP on Rab5-early endosomes to regulate axonal trafficking. Our earlier observations have shown that RIN3 expression (both mRNA and protein) was increased in both cultured E18 BFCNs and 3-month-old brain lysates in APP/PS1 mice (Figs. 1 and 2). To define if BIN1 and CD2AP expression were also altered in APP/PS1 mice [49, 50], we performed immunoblotting with specific antibodies against BIN1 or CD2AP using brain lysates of 3 month-old APP/PS1 mice and their age- gender matched WT mice. Interestingly, we found the neuronal variant of BIN1(Aphiphysin1) was significantly reduced with a concomitant increase in the level of non-neuronal BIN1 isoforms in the APP/PS1 brain compared to WT brain (Fig. 7a, b). For CD2AP, we saw a 2-fold increase in the APP/PS1 mice compared to WT mice (*p* = 0.0184) (Fig. 7a, b). Together, the data have demonstrated a significant alteration in the levels/activities of the RIN3/BIN1/CD2AP complexes in APP/PS1 mouse brains: an increase in both RIN3 and CD2AP with a concomitant reduction in BIN1.

To further investigate developmental changes in the RIN3/BIN1/CD2AP complex, we dissected the cortex and the hippocampus from 10-month-old APP/PS1 mice and age-matched WT mice. The samples were analyzed by immunoblotting (Fig. 7). Intriguingly, both forms of BIN1 showed a decrease in the cortex (Fig. 7c, e) but an increase in the hippocampus (Fig. 7d, f) in APP/PS1 as compared to WT. CD2AP was also significantly upregulated in the hippocampus but not in the cortex in APP/PS1 mice (Fig. 7d, f versus Fig. 7c, e). Consistently, RIN3 was increased in both regions in APP/PS1 mouse brain. We further examined the protein level of Rabex5, another potential GEF for Rab5. In contrast to RIN3, our results did not show an increase in the protein level of Rabex5 (Fig. 7c-f). We also did not see an increase in the protein level of Rab5 (Fig. 7c-f). Together with our earlier results (Figs. 1 and 2), these data have provided evidence that RIN3 is consistently upregulated in APP/PS1 mice.

Recent evidence has suggested a role of BIN1 and CD2AP in APP trafficking processing to regulate extracellular Aβ deposition [51, 52]. BIN1 has been shown to control axonal Aβ production: depletion of the neuronal BIN1 variant disrupted APP recycling process and increased Aβ production within intraneuronal compartments [52]. On the other hand, CD2AP depletion induced APP accumulation in early endosomes that retarded APP degradation to enhance intraneuronal Aβ generation as well [52].

**Fig. 8:**
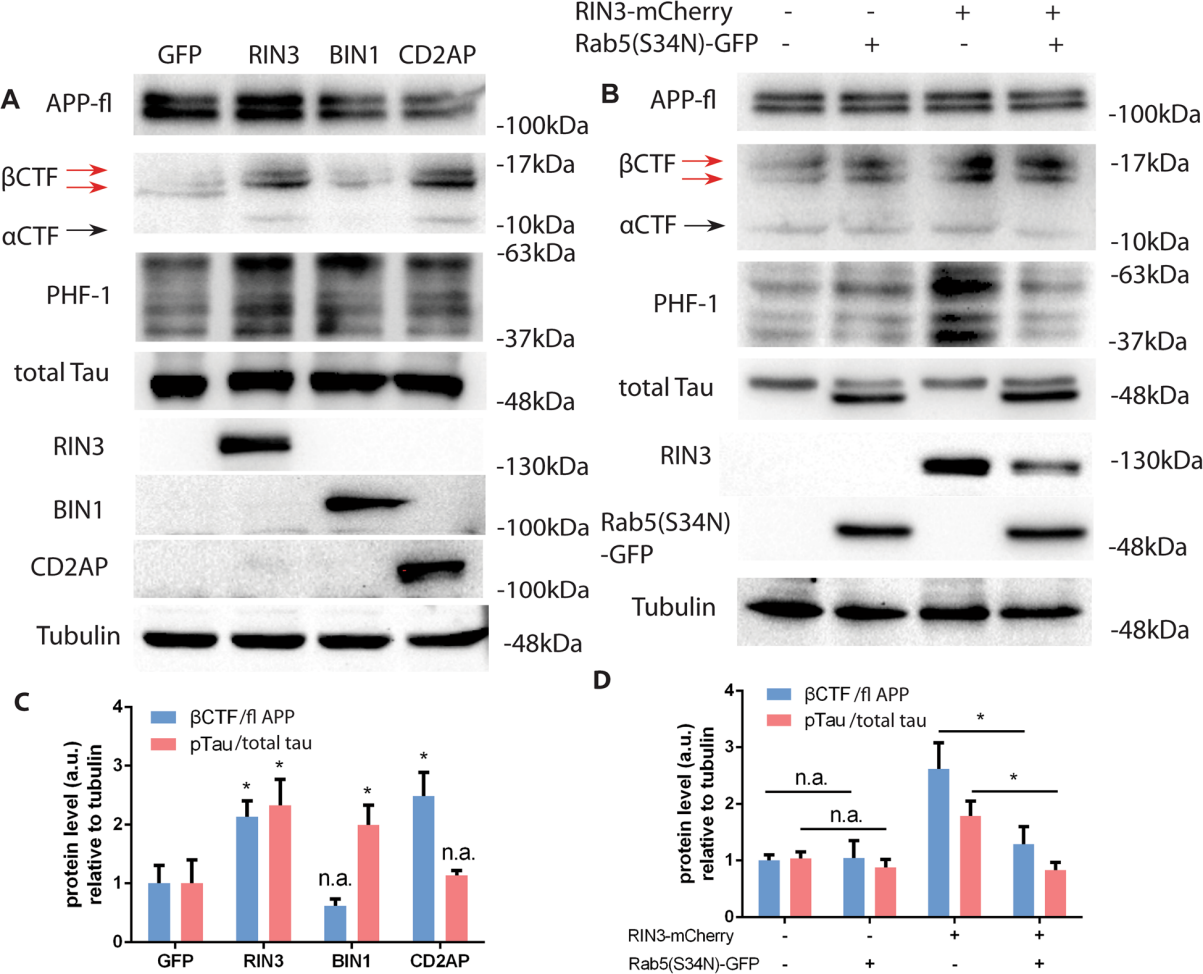
RIN3 promotes APP CTFs production and Tau phosphorylation via Rab5. PC12M cells were transfected with GFP, RIN3-GFP, BIN1-GFP, CD2AP-GFP and cell lysates were immunoblotted using indicated antibodies (**a**). In **b**, PC12 cells were transfected with indicated vector(s) (RIN3-mCherry, Rab5S34N-GFP). and cell lysates were analyzed by SDS-PAGE/immunoblotting with either the APP C-terminals antibodies to detect full length APP/APP CTFs and other antibodies as indicated. Full-length APP (fl APP), APP CTFs, total Tau and phosphorylated Tau are shown (**a** and **b**). Both the ratio of CTFs/APP-fl and the ratio of pTau/total Tau is shown in **c** and **d**. *p* < 0.05 (*), *p* > 0.05 (n.s.), standard t-test

To define the effect of RIN3, BIN1, CD2AP in APP processing and Tau phosphorylation, we expressed these constructs tagged with GFP in PC12 cells. GFP was used as a control. Cell lysates were immunoblotted with a rabbit antibody against the APP C-terminal 15 residues (C15). The C15 antibody recognizes the full length APP as well as various forms of APP C-terminal fragments (APP-CTFs) such as βCTF and αCTF. Consistent with earlier studies [51], upregulating BIN1 had little effect on the level of βCTFs production (Fig. 8a and c). Expression of either RIN3 or CD2AP yielded more βCTFs in PC12 cells (Fig. 8a and c). We detected at least two bands for βCTF, that likely corresponded to either C99 or C99 and its phosphorylated form, as reported by other investigators [53]. When probed the lysates for pTau with the PHF1 antibody, our results showed that expression of both BIN1 and RIN3, but not CD2AP, induced an increase in pTau (Fig. 8a, c).

To further investigate if the effect of RIN3 on the increase in βCTFs and pTau was mediated by Rab5 activation, we transfected a dominant negative Rab5 construct (Rab5^S34N^) into PC12 cells. Expression of Rab5^S34N^ effectively blocked the increase of both βCTFs and pTau induced by RIN3 overexpression (Fig. 8b and d). Of note, although expression of Rab5^S34N^ appeared to result in the appearance of a lower molecular species of Tau, expression of Rab5^S34N^ alone did not alter the level of pTau (Fig. 8b). However, we did observe the appearance of a second Tau species with a lower molecular mass in these Rab5^S34N^-expressing cell samples. To further address the influence of Rab5^S34N^ on total Tau level, we respectively transfected GFP-Rab5^WT^ and GFP-Rab5^S34N^ in PC12 cells using GFP as a control. Total Tau proteins were detected with the same antibody. As shown in Supplementary Figure 5, it turned out that GFP-Rab5^WT^ and GFP-Rab5^S34N^, but not GFP itself, induced the appearance of this lower molecular species of Tau. Therefore, it is likely that overexpression of Rab5 promotes cleavage of Tau. Taken together, these data have demonstrated that the RIN3/BIN1/CD2AP complex may have a dual role in regulating APP processing and Tau phosphorylation; The RIN3/CD2AP arm enhances Rab5 activation and thus to affect APP processing that favors the production and accumulation of neurotoxic βCTFs, while the RIN3/BIN1 axis may affect Tau phosphorylation.

## Discussion

Although increasing genetic evidence has uncovered RIN3 as an AD risk factor, presently, little is known about its exact role in cellular pathogenesis in either LOAD or EOAD. Here we have demonstrated that RIN3 was significantly upregulated in APP/PS1 mouse model of AD. Enhanced expression of RIN3 correlated with enlarged Rab5 early endosomes in primary cultured E18 BFCN neurons from APP/PS1 mice. We have shown that RIN3 interacted with and recruited BIN1 and CD2AP to Rab5 early endosomes. We further demonstrated that by interacting with CD2AP, RIN3 regulated APP trafficking and processing and increased expression of RIN3/CD2AP enhanced the production of neuronal toxic APP βCTFs. On the other hand, through binding to BIN1, increased expression of RIN3 enhanced Tau phosphorylation. The effect of increased expression of RIN3 on both the production of βCTFs and pTau increase is likely mediated by activating Rab5. Our current study has thus provided novel insights into the cellular and molecular mechanisms by which increased RIN3 expression contributes to cellular and neuronal dysfunction that may contribute importantly to early pathogenesis in AD.

Following the initial discovery of RIN3 association with LOAD in a large scaled GWAS in 2011 [13], genetic variant RIN3 was identified in a prospective cohort study of 74,754 individuals together with BIN1 and CD2AP to be associated with increased risk of Alzheimer’s disease, all dementia, and suggested vascular dementia independent of the strong APOE ε4 allele [54]. A recent study enrolled cognitively healthy centenarians as extreme controls suggested that compared to age-matched controls, the effect size increase was significant for *RIN3* (4.5-fold), even more significant than APOE-ε2(2.2-fold) and APOE-ε4(2.0-fold) [22]. This conclusion indicated that the LOAD-RIN3 variant is highly correlated with cognitive function. In addition, a 2017 GWAS defined a missense mutation in RIN3 (W63C) is associated with sporadic EOAD [23]. Therefore, strong evidence supports a role of RIN3 in AD pathogenesis.

In a gene methylation profiling study of blood and brain samples from 22 AD and 26 normal control subjects (27 males, 21 females), AD samples showed significant group-wide hypomethylation of 7 CpGs located within the 3’UTR of RIN3 (CpG1 p  =  0.019, CpG2 p  =  0.018, CpG3 p  =  0.012, CpG4 p  =  0.009, CpG5 p  =  0.002, CpG6 p  =  0.018, and CpG7 p  =  0.013, respectively). The effect was specific since other LOAD risk factors (PTK2β, ABCA7, SIRT1, or MEF2C) did not show significant changes in methylation of their promoters [24]. Hypomethylation is believed to result in enhanced expression of the perspective gene [55]. Consistent with these studies, we detected that RIN3 expression was consistently upregulated in APP/PS1 mice even as early as in cultured E18 BFCNs. The increase in RIN3 expression precedes the appearance of both Aβ-amyloid-containing neurotic plaques and phosphorylated Tau-containing neurofibrillary tangles in APP/PS1 mice with the appears after 3 months of age while the latter becomes visible after 4 months of age [56]. Based on these observations, we speculate that upregulation of RIN3 expression is an early event in AD pathogenesis and that RIN3-induced endocytic dysfunction may play a crucial role in the initiation of early cellular and neuronal events that leads to AD pathogenesis.

Our study has also revealed that RIN3 interacted with BIN1 and CD2AP. BIN1 interacts with the N-terminal region (amino acids 1–586) encompassing the SH3 domain of RIN3 [26]. CD2AP may bind via its SH3 domain to the two PRD domains (367-390aa, 445-462aa) in RIN3 [47]. We confirmed these interactions in our genetic deletion experiments: removal of SH2, RH, Vps9 or the Ras domain on RIN3 didn’t disrupt colocalization of RIN3 with BIN1 and CD2AP. However, without these domains, the RIN3/BIN1/CD2AP complexes failed to be recruited to early endosomes. We thus speculate that RIN3 acts as a scaffold to tether BIN1 and CD2AP specifically to early endosomes.

Amyloidogenic cleavage of APP to yield Aβ likely occurs predominantly in the intracellular compartments [20, 57–59] Under normal conditions, early endosomes marked by Rab5 are a major site of APP processing by β-secretase to yield βCTFs [18, 19]. βCTFs is further processed in late endosomes or trans-Golgi network (TGN) to give rise to Aβ. The neuronal toxicity of βCTF has been observed in multiple studies. Recent studies have pointed to strong adverse impact of βCTF on synaptic plasticity and neuronal function and is intimately linked to early cellular pathology, independent of Aβ, in AD [14, 18, 21, 53, 60–63]. Axonal APP was more likely to undergo cleavage and produce Aβ. Accordingly, we found overexpressed RIN3 in primary cortical neurons impaired APP and BACE1 trafficking by increasing stationary vesicles and slower trafficking velocities, and upregulating RIN3 led to increased APP CTFs production. This effect was rescued by Rab5^S34N^, supporting that increased RIN3 promoted APP CTFs in early endosomes.

Unlike RIN3, BIN1 and CD2AP have been extensively studied for their function in regulating endocytic trafficking and their role in AD pathogenesis [20, 51, 52, 64]. CD2AP, a membrane-associated scaffolding protein, likely controls the assembly of protein complexes, participates in endocytosis and endocytic trafficking to transmit intracellular signals [45]. It was shown that CD2AP, by virtue of its multiple protein-protein binding modules, interacts with multiple proteins involved in diverse biological processes [45]. Previous study has shown that suppression of CD2AP resulted in a decreased Aβ_42_/Aβ_40_ ratio both in N2a neuron-like cell lines and in 1 month-old APP/PS1 mice [65]. And deletion of CD2AP also decreased APP CTFs [52]. Consistent with these studies, our results show that increased CD2AP compromised APP and BACE1 trafficking, leading to increased APP-CTFs.

BIN1, also known as amphyphisin II, has 10 isoforms. The longest isoforms expressed predominantly in central nerve system contains a unique clathrin-AP2 binding region (CLAP), while the shorter isoforms are ubiquitous [11]. And our data shown that the brain-specific isoform level was decreased in 3 months old APP/PS1 mice, while the ubiquitous isoform level was increased. Although the significance is presently unknown, the changes in expression of BIN1 isoforms have also been recently reported in human postmortem brain samples when comparing healthy control to AD patients [11, 50, 64, 66, 67]. Although the role of BIN1 in regulating APP processing is currently under debate [51, 52, 68, 69], BIN1 has been found to interact with Tau [51, 64, 68, 69], indicating its potential role in regulating Tau biology. This is confirmed in a recent study that increased BIN1 expression was found disrupting their eye morphology by modulating Tau pathology in *Drosophilia* rather than Aβ42 pathology [68]. Consistent with these findings, we have demonstrated that RIN3/BIN1 overexpression led to upregulated hyperphosphorylated Tau. Although the role of BIN1 in regulating Rab5 early endosome is unclear, a recent study have showed that conditional knockout of BIN1 also induced enlargement of Rab5 endosomes in mice [70]. It is possible that the loss of BIN1 in this case could alter the balance between RIN3/BIN1 and RIN3/CD2AP that frees more RIN3 to activate Rab5. Nevertheless, future studies are needed to define the exact role of the two RIN3 complexes (RIn3/CD2AP, RIN3/BIN1) in regulating neuronal functions.

Our current study has pointed to a novel but intriguing role for RIN3 in early AD pathogenesis: By interacting with CD2AP in early endosomes, RIN3 participates in APP trafficking and processing. Increased expression of RIN3 impairs APP trafficking and enhances APP cleavage; By recruiting BIN1 to early endosomes, increased RIN3 expression also promotes Tau hyperphosphorylation. As such, RIN3 contributes importantly to early cellular pathology in AD pathogenesis. Future in vivo studies will be needed to define and validate this novel hypothesis. Since RIN3, BIN1, CD2AP are also expressed in microglial cells [71], it is also vitally important to define the role of RIN3 and the RIN3/BIN1/CD2AP complexes in processes such as Aβ clearance by microglia in the future as well.

## Conclusion

In summary, we have defined and uncovered an important role for RIN3 in AD pathogenesis. We have shown that increased expression of RIN3 in early stages of AD pathogenesis that results in enlarged Rab5 endosomes. We have also demonstrated that RIN3 recruited two other AD risk factors BIN1 and CD2AP to Rab5 early endosomes. Through Rab5 activation, increased RIN3 and CD2AP impaired trafficking and processing of APP leading to the generation of neuronal toxic APP-CTFs. The increase in the RIN3/BIN1 complex may induce pTau. Together, our study has provided exciting insights into the potential cellular mechanisms by which upregulation of RIN3 contributes to early cellular pathology in early stages of in AD pathogenesis.

## Supplementary information

**Additional file 1: Figure S1.** BIN1 and CD2AP colocalize with different RIN3 variant. Different RIN3-GFP constructs were co-transfected into PC12M cells with either CD2AP-flag or BIN1-flag, followed by immunostaining for the flag tagged constructs or Rab5 using specific antibodies. Representative images are shown.

**Additional file 2: Figure S2.** BIN1 and CD2AP are not recruited to Rab7 late endosome by RIN3. PC12M cells expressing GFP/CD2AP-flag (A, B), RIN3-GFP/CD2AP-flag (C, D), GFP/BIN1-flag (E, F), RIN3-GFP/BIN1-flag (E-H) were immunostained for Rab7 with a specific antibody. Representative images are shown. Colocalization analysis was performed by ImageJ by measuring fluorescence intensity alongside the drawn line. The three different colors peak at the same position If the three proteins were colocalized (B, D, F, H).

**Additional file 3: Figure S3.** BIN1 and CD2AP are not recruited to Rab11 recycling endosome by RIN3. PC12M cells expressing GFP/CD2AP-flag (A, B), RIN3-GFP/CD2AP-flag (C, D), GFP/BIN1-flag (E, F), RIN3-GFP/BIN1-flag (E-H) were immunostained for Rab11 with a specific antibody. Representative images are shown. Colocalization analysis was performed by ImageJ by measuring fluorescence intensity alongside the drawn line. The three different colors peak at the same position If the three proteins were colocalized (B, D, F, H).

**Additional file 4: Figure S4.** Colocalization of RIN3, BIN1 and CD2AP with APP. APP-mCherry was co-transfected into mouse E18 primary cortical neurons with either GFP, or RIN3-GFP or BIN1-GFP or CD2AP-GFP (A). Representative images are shown. In B, representative images of neurites are shown. C. Results for semiquantitative analyses for colocalization are shown.

**Additional file 5: Figure S5.** Overexpression of Rab5 induces cleavage of Tau. GFP, GFP-Rab5^WT^, GFP-Rab5^S34N^ (dominant-negative form) expression vectors were transfected in PC12 cells as indicated. Cells were harvested and lysates were analyzed by SDS-PAGE/ immunoblotting with an antibody against total Tau. GFP antibody was used to detect transfection efficiency and GAPDH was used as endogenous control.

**Additional file 6: Table 1.** Identify RIN3 interacting protein via Mass Spectrum. Recombinant flag tagged RIN3 protein were purified from HEK293T cells. RIN3-interactomes were identified by Mass Spectrometry. Two replicates were performed. PSM representing abundancy of each protein were used to sort the proteins. BIN1 and CD2AP were highlighted with light yellow in tables.

## Data Availability

The datasets used and/or analyzed during the current study are available from the corresponding author on reasonable request.

## Abbreviations

RIN3: Ras And Rab Interactor 3
BIN1: Bridging Integrator 1
CD2AP: Cluster of differentiation 2 Associated Protein
APP: Amyloid precursor protein
PS: Presenilin
NTFs: Neurofibrillary tangles
CTF: Carboxyl terminal fragment
GWAS: Genome wide association study
CLU: Clusterin
CR1: Complement Receptor 1
PICALM: Phosphatidylinositol binding clathrin assembly protein
ABCA7: ATP-binding cassette transporter A7
MS4A4: Membrane-spanning 4-domains subfamily A
EPHA1: Eph receptor A1
CD33: Cluster of differentiation 33
INPP5D: Inositol polyphosphate-5-phosphatase
MEF2C: Myocyte enhancer factor 2C
HLA-DRB1/HLA-DRB5: the Major Histocompatibility Complex Class II, DRβ1 and 5 NME8
ZCWPW1: Zinc finger, CW type with PWWP domain 1
PTK2B: Protein tyrosine kinase 2β
SORL1: Sortilin related receptor L1
CELF1: CUGBP, elav-like family member 1
SLC24A4: Sodium/Potassium/Calcium Exchanger), Member 4
FERMT2: Fermitin Family Member 2
CASS4: Cas Scaffolding Protein Family Member *4*
GEF: Guanine nucleotide exchange factor
LOAD: Late onset AD
EOAD: Early onset AD
sEOAD: Sporadic EOAD
BFCNs: Basal forebrain cholinergic neurons
WT: Wild Type
APOE: Apolipoprotein E
BACE: β-secretase
TGN: Trans-Golgi network
SH2: Homology 2 (SH2)
*TrkA*: *Tropomyosin receptor kinase A*
ChAT: Choline acetyltransferase
