# Supplementary figures and images for: Upregulation of RIN3 induces endosomal dysfunction in Alzheimer’s disease
Source: Transl Neurodegener. 2020 Jun 18;9:26. doi: 10.1186/s40035-020-00206-1 (PMC7301499; doi:10.1186/s40035-020-00206-1)

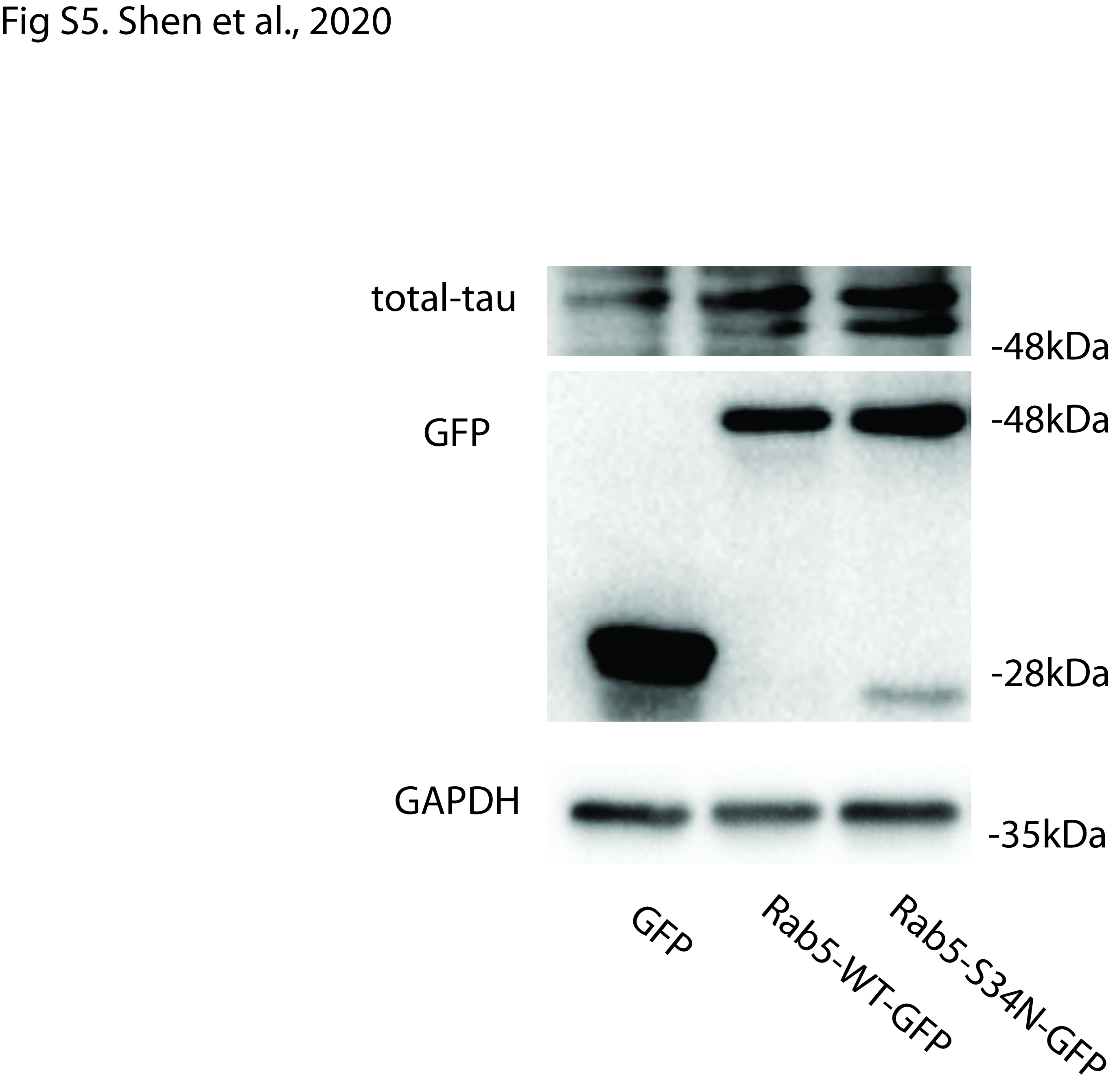

Supplement: Supplementary file 5 — Additional file 5: Figure S5. Overexpression of Rab5 induces cleavage of Tau. GFP, GFP-Rab5WT, GFP-Rab5S34N (dominant-negative form) expression vectors were transfected in PC12 cells as indicated. Cells were harvested and lysates were analyzed by SDS-PAGE/ immunoblotting with an antibody against total Tau. GFP antibody was used to detect transfection efficiency and GAPDH was used as endogenous control. [file 40035_2020_206_MOESM5_ESM.tif]
